# Anemia in Heart Failure Patients

**DOI:** 10.5402/2012/246915

**Published:** 2012-03-25

**Authors:** Michael G. Alexandrakis, George Tsirakis

**Affiliations:** Department of Hematology, University Hospital of Heraklion, P.O. Box 1352, 71110 Heraklion, Crete, Greece

## Abstract

Heart failure is a very common disease, with severe morbidity and mortality, and a frequent reason of hospitalization. Anemia and a concurrent renal impairment are two major risk factors contributing to the severity of the outcome and consist of the cardio renal anemia syndrome. Anemia in heart failure is complex and multifactorial. Hemodilution, absolute or functional iron deficiency, activation of the inflammatory cascade, and impaired erythropoietin production and activity are some pathophysiological mechanisms involved in anemia of the heart failure. Furthermore other concomitant causes of anemia, such as myelodysplastic syndrome and chemotherapy, may worsen the outcome. Based on the pathophysiology of cardiac anemia, there are several therapeutic options that may improve hemoglobin levels, tissues' oxygenation, and probably the outcome. These include administration of iron, erythropoiesis-stimulating agents, and blood transfusions but still the evidence provided for their use remains limited.

## 1. Introduction

Cardiovascular diseases are among the most frequent causes of death worldwide [1, 2]. Heart failure is an enormous medical and societal burden and a leading cause of hospitalization. It is estimated that 2.6 millions hospitalizations annually in the USA are due to heart failure as a primary or secondary diagnosis [3].

In the last 19 years the role of several immunological, metabolic, and neurohormonal abnormalities has been recognized in the pathophysiology and progression of the congestive heart failure (CHF) [4, 5]. Among them, anemia and renal failure seem to be major risk factors for an adverse outcome. The cardio renal anemia syndrome (CRAS) represents a pathological triangle in which the primary failing organ is the heart or the kidney and the dysfunction of one organ leads to dysfunction of the other [6]. The presence of anemia or renal dysfunction increases morbidity and mortality in patients with heart failure. It seems that there is an impaired mechanism operating between congestive heart failure, chronic kidney disease (CKD), and anemia, where each might cause or worsen the other. Therefore, correction of anemia would be a major part of this vicious circle in the reduction of the severity of the heart failure [6]. This could be explained by the fact that a significant feature of the congestive heart failure is impaired energy metabolism and therefore the failing heart is an energy-starved heart [7]. Oxygen delivery through hemoglobin (Hb) is essential for energy production and improvement of Hb levels could also improve energy production in cardiomyocytes. Simultaneously energy-sparing treatments may also improve the prognosis [7].

## 2. Epidemiology of Anemia in Heart Failure

Using the historical definition by the World Health Organization, anemia is defined when Hb concentration is less than 13 g/dL for men or less than 12 g/dL for women [8]. However, particularly in the setting of heart failure, this definition has not been subjected to rigorous clinical validation and its appropriateness and clinical applicability continues to be debated [9]. Therefore, some investigators use more conservative definitions (e.g., <12 g/dL for men and <11 g/dL for women) to ensure a higher confidence in capturing the affecting population [10].

Anemia is prevalent in patients with CHF but the exact rates vary widely [11]. A recent meta-analysis of 153,180 patients with CHF, reported in 34 published studies from 2001 to 2007, estimated the prevalence of anemia to be 37.2% (10–49%) [10]. Similarly, the latest prospective STAMINA-HFP (Study of Anemia in a Heart Failure Population) Registry estimated a prevalence of 34% [11]. The variability in the estimated prevalence of anemia is partly attributable to use of different definitions of anemia, whereas patients in the acute decompensated states experience more dilutional anemia and therefore the prevalence may be increased. Patients with CHF and anemia tend to be older than their nonanemic counterparts [12], whereas, in patients less than 55 years, the age of anemic and nonanemic patients does not appear to differ [13]. Concerning the gender, in studies of CHF and anemia enrolling a preponderance of men, the proportion of women steadily increases as Hb concentration falls to the point that women can predominate among patients with CHF and severe anemia [14].

One of the most frequent comorbid conditions in patients with CHF is CKD (as defined by an estimated glomerular filtration rate (eGFR) <90 mL/min^−1^/1.72 m^−2^). CHF and CKD share some common causes (e.g., hypertension), features (e.g., malnutrition, impaired performance status), and risk factors (e.g., older age). In a meta-analysis of 16 studies, it was found that 63% of 80,098 patients with CHF had some degree of concomitant impaired renal function and 29% of them had severe CKD. This is associated with an increased risk of adverse outcome [15], being probably a stronger predictor of mortality than ejection fraction (EF) or New York Heart Association (NYHA) functional classes [16]. Anemia is more prevalent when CHF and CKD coexist in both ambulatory and hospitalized settings [17]. In large CHF registries the degree of anemia closely parallels to eGFR, although primary renal disease is relatively uncommon in CHF [17, 18]. This justifies that kidneys play a major role in the pathophysiology of anemia in CHF. It is important that patients with CHF and CKD develop anemia in higher values of eGFR than patients with CKD alone. This provides indirect evidence that other factors than CKD are involved in the pathophysiology of anemia of CHF [19].

Anemia exacerbates symptoms of heart failure. There is an impaired mechanism in which tissue hypoxia and release of nitric oxide (NO) cause decreased arteriolar resistance and peripheral vasodilatation. These in turn lead to decreased blood pressure, increased sympathetic activation, renal vasoconstriction, reduced renal function, and activation of renin-angiotensin aldosterone system. The results are production of antidiuretic hormone, fluid retention, left ventricular (LV) hypertrophy and dilation, worsening of heart failure, release of brain natriuretic peptide (BNP), and signs from stress on myocardium. The final outcome, completing the vicious circle, is further anemia. This, however, implies that in the presence of volume overload there might be a decrease in Hb concentration and also oxygen content, although red cell mass remains stable [20]. The patient complains of shortness of breath, tachycardia, dizziness, faintness, and fatigue. Therefore, the presence of anemia is tightly linked to clinical severity of CHF. Symptomatic deterioration and fluid retention inevitably lead to hospitalization. Thus, there is a greater prevalence of anemia in hospitalized patients than ambulatory ones [14]. On the other hand, the presence of more advanced NYHA functional classes has been associated with greater prevalence of anemia [21, 22]. Furthermore, anemic patients with CHF have more commonly diabetes mellitus and more advance disease, with higher NYHA class and more severe symptoms. Those symptoms include lower exercise capacity, worse quality-of-life scores, greater peripheral edema, lower dry weight and blood pressure, higher use of diuretics and other cardiovascular medications, and worse neurohormonal profile (such as renal dysfunction, high BNP and C-reactive protein, low serum albumin) (range, 30% to 61% versus range, 4% to 23% for less symptomatic ambulatory patients) [23, 24]. It is remarkable that anemia does not seem to be related to LV dysfunction, whereas in few studies Hb levels were inversely related to EF. That means that patients with lower values of Hb had higher EF, whereas increase of Hb could decrease LVEF, especially in CKD, in a dose-dependent manner [22, 25–27].

Finally, most studies indicate that the prevalence of anemia is increased in patients with CHF who also have co-morbid kidney disease, advanced age, and more severe symptoms when compared to less symptomatic ambulatory populations [23].

## 3. Pathophysiology of Anemia in Heart Failure

**Figure 1:**
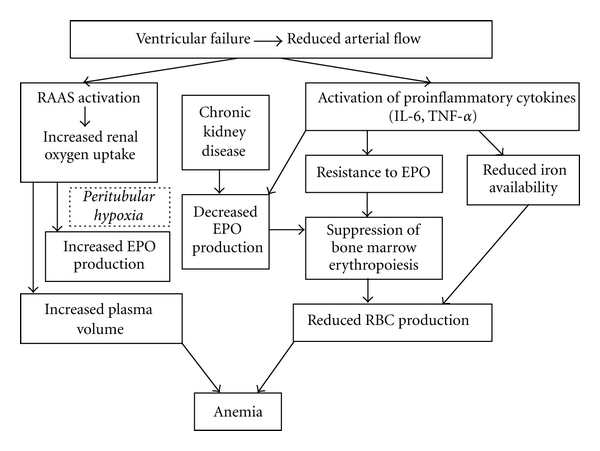
Pathophysiological mechanisms contributing to anemia in chronic heart failure patients. *(RAAS: renin-angiotensin-aldosterone system, IL-6: interleukin-6, TNF-*α*: tumor necrosis factor alpha, EPO: erythropoietin, RBC: red blood cells). *

The major factors contributing to CHF-related anemia involve CKD, renin-angiotensin system, hematinic abnormalities, mainly iron deficiency, chronic inflammation, and hemodilution (Figure 1). 

A major factor contributing to anemia of CHF is kidney dysfunction, being associated with the cardiac disorder. The renal damage in the CHF is mainly hypoxic, due to reduced renal flow, caused by the reduced cardiac output [14, 24]. Hypoxia induces erythropoietin (EPO) production by peritubular fibroblasts, although renal blood flow in CHF is relatively maintained until the late stages of the syndrome, especially when receiving angiotensin-converting enzyme (ACE) inhibition. Nevertheless, EPO production does not seem to correlate with effective renal plasma flow. It only correlates weakly with eGFR. This suggests that renal dysfunction plays a role in the blunted EPO production in anemic patients with CHF [28], resulting in increased EPO levels but not as expected for the degree of anemia, suggesting that in CHF there is both blunted EPO production and resistance to EPO [28]. Furthermore, the coexistence of CHF with CKD is associated with reduced EPO production from the kidney [20], as well as with urinary losses of serum EPO and transferrin [29, 30], which further deteriorate the anemia.

The renin-angiotensin system seems also to be involved in the control of erythropoiesis. Angiotensin II reduces renal blood flow, increases the oxygen demands, and thereby stimulates EPO production [24]. It also stimulates the proliferation of normal bone marrow early erythroid progenitors in a direct manner [31]. Both ACE inhibition and angiotensin receptor blockade decrease erythropoiesis, causing a modest reduction in Hb, up to 0.3 g/dL [14, 32]. This suppression is attributed to a mild reduction of EPO production and also to prevention of hematopoiesis inhibitor N-acetyl-seryl-aspartyl-lysyl-proline (AcSDKP) breakdown. AcSDKP is normally degraded by the amino terminal catalytic domain of ACE. ACE inhibition should be expected to cause a mild reduction of erythropoiesis, although various knockout mice models, involving different ACE components, do not support this theory [33].

Iron deficiency is common in patients with CHF especially when accompanied by CKD [34], whereas vitamin B12 and folic acid deficiencies or iron overload are not. It is of interest that the incidence of iron deficiency is increasing with the severity of heart failure [35]. In half cases, iron deficiency is absolute (with low transferrin saturation and serum ferritin, usually associated with decreased iron stores and reduced iron deposits in the bone marrow). In the other half cases iron deficiency is functional-relative (with low transferrin saturation and normal or elevated serum ferritin, usually associated with normal or elevated iron stores and iron deposits in the bone marrow) [36]. It has been reported that in 17% of anemic patients with CHF the iron deficiency is both absolute and functional [35].

There are many causes of absolute iron deficiency associated with CHF, especially in coexistence with CKD. These include

1. low iron intake (due to low protein diets and anorexia),
2. gastrointestinal blood loss (due to platelet dysfunction or coagulation abnormalities; caused by platelet inhibitors, anticoagulants, or uremia),
3. iron malabsorption (due to either CHF or uremia related-bowel edema, causing intestinal cell dysfunction, or to proton pump inhibitors or to phosphate binders, that also bind iron),
4. removal of blood for tests.

In CHF the functional iron deficiency is related to iron disuse, resembling anemia of chronic disease, as evidenced by iron acquisition by the reticuloendothelial system [37]. In patients with severe CHF, elevation of several inflammatory cytokines serum levels has been found. Among them, interleukin-1 (IL-1), interleukin-6 (IL-6), tumor necrosis factor-*α* (TNF-*α*), and less frequently interleukin-18 (IL-18) seem to be the most important, whereas IL-6-induced hepcidin expression also participates in the phenomenon [14, 38, 39]. This inflammatory process causes reduced EPO production, through activation of GATA 2 binding protein and nuclear factor-*κ*B, and impaired response to bone marrow erythroblasts. It also causes hepcidin-induced blockade of iron absorption from the gut and iron trapping in reticuloendothelial system's stores. Hepcidin is an acute phase antibacterial protein, induced by IL-6, through JAK/Stat3 pathway, released from the liver and excreted from the urine. Therefore, in CHF with a concomitant CKD, there is a reduced hepcidin removal from the kidney, implying a further increase of its levels. Hepcidin inhibits ferroportin, a protein expressed on intestinal cells, macrophages, and hepatocytes that releases the iron from those cells into the blood. Hepcidin-induced inhibition of ferroportin causes decreased iron absorption from the gut and blockade of iron release from its stores, in hepatocytes and macrophages, into the blood. This implies inadequate iron delivery to the bone marrow erythroblasts, although the total stores may be adequate, causing a functional iron deficiency. Furthermore hepcidin seems to exert a direct inhibitory effect on erythroblast proliferation and survival [40].

Iron metabolism is crucial for energy production in the body and most importantly for cells with high energy demands, such as cardiomyocytes [34]. Iron plays a crucial role in oxygen transportation (as a component of Hb), oxygen storage (as a component of myoglobin), oxidative metabolism (as a component of oxidative enzymes and respiratory chain processes), and in metabolism of lipids, carbohydrates, nucleic acids, collagen, tyrosine, and catecholamines [41, 42]. In CHF, an energy-starved situation, several disorders of iron metabolism have been observed. Iron deficiency, absolute or functional, can impair oxidative metabolism, cellular energetic, and cellular immune mechanisms. Iron deficiency in rat hearts causes mitochondrial ultrastructural aberrations, irregular sarcomere organization, and release of cytochrome C [43]. In addition, experimental animal models with severe iron deficiency have major disruption in energy production causing cardiac damage, with diastolic dysfunction and heart failure, accompanied by reduced EPO and increased TNF-*α* serum levels and worsening of molecular signaling pathways. Those defects may participate in the transition from adaptive cardiac hypertrophy to permanent cardiac impairment in chronic iron deficiency [44]. On the other hand, iron seems to have anti-inflammatory effects. It has been shown that hemodialysis patients receiving EPO with IV iron supplementation had lower inflammation markers (lower levels of proinflammatory TNF-*α* and free radicals, as expressed by total peroxides, and higher levels of anti-inflammatory interleukin-4) compared to patients receiving EPO alone [45]. Furthermore iron deficiency anemia seems to enhance red cell oxidative stress [46] and has been associated with lower peak oxygen consumption and higher ratios of ventilation to carbon dioxide production [35]. In a recent study the disordered iron homeostasis has been identified as an independent risk factor for death [35]. 

Another effect of iron deficiency on CHF patients is the consequent thrombocytosis. CHF is a hypercoagulable state [47], where the co-existence with iron deficiency-related thrombocytosis [48] increases the risk of thrombosis and the mortality rate [49]. Furthermore, it has been shown that the concomitant administration of IV iron with EPO (which can cause iron deficiency) in hemodialysis patients significantly reduces the platelet counts, compared to patient receiving EPO alone [50]. It has also been shown that the use of erythropoiesis-stimulating agents (ESAs) in iron-deficient patients increases the risk of thrombocytosis and thrombosis [51]. 

Except iron disuse, inflammatory cytokines may cause EPO resistance in anemic CHF patients. It has been suggested that there is a diminished responsiveness of erythroid cells to EPO, being accompanied by increased levels of inflammatory cytokines, such as IL-6, soluble TNF receptor 2, and TNF-*α* levels [52, 53]. Their activation does not result in EPO receptor downregulation, but in blunted EPO-induced JAK-STAT signaling [52]. This is confirmed by the partial abrogation of the inhibitory effects of anaemic sera on erythroid colony growth by anti-TNF-*α* neutralising antibodies [53]. Furthermore, IL-6-induced hepcidin exerts a direct inhibitory effect on erythroblast proliferation and survival [40].

Another factor contributing to anemia of CHF patients is NO. Endothelial dysfunction associated with heart failure may alter endothelial NO synthase activity, hence further augmenting myocardial dysfunction due to increased oxidative stress [54]. Paralleling these cardiodepressive actions, NO seems to have a direct inhibitory effect on bone marrow hematopoietic activity [55, 56]. Furthermore, it is of interest that NO inhibits blood cell formation in nonischemic murine CHF, whereas inflammatory cytokines, such as TNF-*α*, impair hematopoiesis in CHF following myocardial infraction [57].

A recent study suggests that vitamin D deficiency is independently associated with anemia in end-stage heart failure, based on the fact that vitamin D may stimulate erythropoiesis. On this study circulating 1,25-dihydroxyvitamin D was a better predictor of anemia than circulating 25-hydroxyvitamin D. Prospective randomized studies with administration of vitamin D will have to clarify if the association of vitamin D deficiency with anemia is causal [58].

In conclusion anemia in CHF is multifactorial. Two major factors contributing and exacerbating to its appearance are kidney dysfunction and iron deficiency. In 2003 this abnormality was described as cardio renal anemia syndrome (CRAS) [6], whereas the correction of anemia could play a major role in this vicious circle in improving the severity of CHF. In the last years, the role of iron has been recognized, as a major component of many energy-producing systems. In view of a possible independent association of iron deficiency and cardiac failure, renal failure and anemia, the same authors rename the syndrome as cardiorenal anemia iron deficiency (CRAID) syndrome [6].

## 4. Treatment of Anemia in Heart Failure

CHF is not just a hemodynamic disorder. It is the final common pathway of other conditions, where renal failure and anemia contribute to the progression to a more severe disease status. They also could be potential targets for intervention. Since the two major components of CHF anemia are iron deficiency and reduced EPO activity (absolute or functional in both cases), the main goals of intervention would be to increase their levels. Anemia treatment strategies in heart failure patients include erythropoiesis-stimulating agents (ESAs) and red blood cell transfusions. Iron replacement in iron-deficient patients with or without anemia has also been investigated. Before starting any treatment for anemia in CHF, it is necessary to exclude and treat, if possible, any other causes of anemia, such as active bleeding, hemolysis, vitamin B12 or folate deficiency, or even more chronic situations such as myelodysplastic syndromes and other malignancies. 

Health Services Research & Development Service's Evidence-based Synthesis Program has collected the literature from 1949 until November 2010 and published a review regarding the treatment of anemia in CHF and coronary heart disease patients [59]. Despite the association with poorer outcomes, it remains unclear whether treating anemia or iron deficiency may improve outcomes.

It has been suggested that a small reduction of Hb levels may worsen the outcomes and symptoms of CHF, whereas the correction of anemia may improve NYHA classification, LVEF, LVH, and diuretic response [60–63]. Treatment has been centered on administration of erythropoiesis-stimulating agents (ESAs) and parenteral iron supplementation, but most studies are poorly powered and therefore with limited validity. On the other hand, the only way to cause a rapid increase of Ht and therefore tissue oxygenation is the blood transfusion. Nevertheless, the only recommendations referring to anemia of CHF suggest treating any correctable causes of anemia, if this is evitable, such as iron, folate, or vitamin B12 deficiencies [64]. The goal of Hb correction is also not well defined. Transfusion of packed red cells in Hb values lower than 9 g/dL or Ht less than 30% may be suggested, but in the setting of acute coronary syndromes this has been associated with higher mortality [65].

There are many data supporting that iron deficiency may contribute to the increased mortality in CHF patients. It has also been shown that correction of iron deficiency could improve symptoms and status of the syndrome. Nevertheless, this has not been adequately confirmed [34]. There are several studies in CHF, with iron deficiency, with and without anemia, suggesting that correction of iron deficiency, using IV preparations, could improve symptoms and signs of CHF, such as S′-wave, E/E′ ratio, peak systolic strain rate, NYHA class, 6-minute walk distance, even without improvement of EF or rise of Hb levels, without major side effects [66–70]. It is suggested, with high evidence, that correction of iron deficiency can improve exercise tolerance and duration, as well as the quality of life, in patients with stable CHF and mild CKD [59]. There are also other studies suggesting that anemia in CHF can be corrected only with IV iron and not the oral forms [71–74], whereas long-term oral iron does not seem to improve any CHF parameters [75, 76]. This seems reasonable since in CHF there is increased hepcidin expression that blocks iron use, even if it is absorbed. In general terms it is preferred to administrate IV instead of oral iron, even though it can cause oxidative stress. When using intravenous preparations, the majority of the dose is deposited in long-term storage. There is a small portion of this iron that is rapidly bound to transferrin and available for transport to the bone marrow, bypassing the restrictions on iron release imposed by hepcidin. The use of large amounts of iron, delivered over minutes or hours as pulse therapy, could lead to poor utilization of this iron with tissue deposition, free radical formation, and increased risk of infection, because of a decrease in cellular immunity and promotion of bacterial growth [69]. Other concerns of iron administration have to do with an increased risk of coronary heart disease, but still have not been confirmed [34]. In conclusion, there are not efficient data proving evidence that iron administration in non-iron-deficient patients with CHF could improve the cardiac disease. It is however of great value to recognize iron deficiency, even in the absence of anemia and to correct it.

Regarding ESAs, there are only pilot studies for their use in the treatment of anemia of CHF. Uncontrolled hypertension and iron deficiency are contraindications. They are commonly used with simultaneous administration of IV iron, giving promising results in ameliorating symptoms and improving cardiac function, but only with very limited evidence [77, 78]. Moreover, the EPO receptor is present in many nonerythropoietic tissues, including myocardium, endothelium, vascular smooth muscle cells, and neurons, where EPO has shown tissue protective properties, because of its antiapoptotic action [78]. Treatment with EPO in excitable murine and human left ventricular muscle preparations have resulted in an increase in twitch tension and in peak sarcomere shortening. This suggests that EPO exhibits direct positive inotropic and lusitropic effects in cardiomyocytes and ventricular muscle preparation, being mediated through PI3-K and PKC*ε* isoform signalling, to directly affect both calcium release dynamics and myofilament function [79]. Therefore, there could be an extra role of EPO in ameliorating symptoms, in addition to improving Hb levels. The major concern of EPO administration, based on hemodialysis experience, has to do with a possible increase of thrombotic risk, especially by overzealous correction of anemia or when iron-deficiency-associated thrombocytosis coexists. At present, there are not clearly defined targets of Hb in CKD anemia, although an Hb level of 10 g/dL seems to be a widely accepted goal.

Recently, there have been many studies of anemia in CHF patients with an effort to improve Hb levels. There have been used ESA and oral or IV iron and even IV iron without ESA, that have shown a positive effect on hospitalization, NYHA functional class, cardiac and renal function, quality of life, exercise capacity, and reduced BNP, without any increase in cardiovascular damage related to the therapy. However, adequately powered long-term placebo-controlled studies of ESA and IV iron in CHF are still needed and are currently being carried out [80]. Nevertheless, until recently, there is high evidence that the use of ESA in stable CHF patients with serious renal disease did not have any particular effect on cardiovascular events, whereas there is moderate evidence that their use might have a negative impact on the survival [59].

Recent studies have recognized that dilutional anemia is highly prevalent in CHF patients. In this regard, arginine vasopressin antagonists might be an attractive treatment option, by increasing aquaresis. Until now, there is only indirect evidence supporting a rationale for their use. Therefore, future clinical research should explore the role of arginine vasopressin pathway activation in determining dilutional anemia and, ultimately, assess it as a therapeutic target [81].

Anemia is common in patients with heart disease. The evidence base to date does not convincingly support a role for ESAs for anemia correction. On the other hand, iron treatment may help ameliorate symptoms over the short term in patients with symptomatic heart failure. The role of blood transfusions remains understudied and unclear [59].

## 5. Conclusions

It seems that anemia exacerbates CHF, causing a vicious circle, where renal dysfunction and neurohormonal and proinflammatory cytokine activation participate in the development of anemia. On the other hand, anemia increases myocardial workload and worsens cardiac dysfunction. So, it is important to recognize any possible causes of anemia. It would also be beneficial to treat them, if possible. Administration of iron and ESAs seems to be promising, since they can both also improve factors other than anemia, but still there are many questions to be answered. These mainly concern their safety, the goals in Hb elevation, and probably their cost. Further studies are required to understand the association of anemia with CHF outcomes, to recognize the impact of anemia improvement, to asses when to initiate and when to cease the treatment, and finally to estimate the safety of these interventions.

## Abbreviations

ACE: Angiotensin-converting enzyme
AcSDKP: N-acetyl-seryl-aspartyl-lysyl-proline
BNP: Brain natriuretic peptide
CHF: Congestive heart failure
CKD: Chronic kidney disease
CRAID: Cardio-renal-anemia-iron deficiency
CRAS: Cardio-renal anemia syndrome
eGFR: Estimated glomerula filtration rate
EF: Ejection fraction
EPO: Erythropoietin
ESAs: Erythropoiesis stimulating agents
Hb: Hemoglobin
IL-1,6,18: Interleukin-1,6,18
IV: Intravenous
LV: Left ventricular
NO: Nitric oxide
NYHA: New York heart association
TNF-*α*: Tumor necrosis factor-*α*.
